# Supplementary material for: Sex Differences in the Survival of Patients with Neuroendocrine Neoplasms: A Comparative Study of Two National Databases
Source: Cancers (Basel). 2024 Jun 28;16(13):2376. doi: 10.3390/cancers16132376 (PMC11240657; doi:10.3390/cancers16132376)
Supplement: Supplementary file 1 [file cancers-16-02376-s001.zip › cancers-3063225-supplementary.pdf]

Supplementary materials

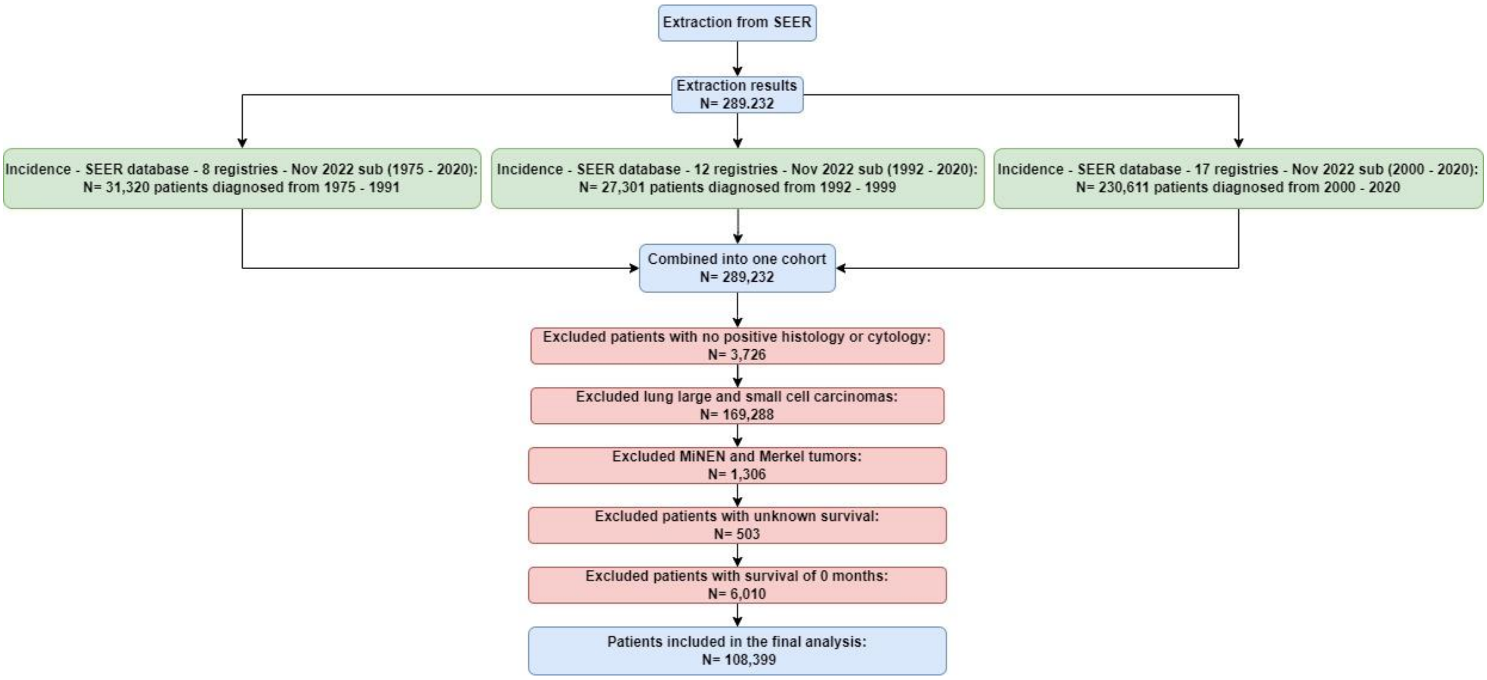

Figure S1. Flowchart detailing stages of data extraction (SEER).

Table S1. Age adjusted female to male 60-months RMTL ratio for patients with NEN in NCRAS and SEER cohorts (classified by sex and site)[1].

Site	sex	Age-adjusted female to male 60-months RMTL ratio	
		RMTL ratio	CI
ALL (UK)	M<br>F	0.71	0.68 – 0.75
ALL (USA)	M<br>F	0.75	0.74 – 0.77
Appendix (UK)	M<br>F	0.71	0.51 – 0.98
Appendix (USA)	M<br>F	0.71	0.70 – 0.72
Cecum (UK)	M<br>F	0.86	0.71 – 1.06
Cecum (US)	M<br>F	0.92	0.92 -0.93
Colon (UK)	M<br>F	1.06	0.93 – 1.21
Colon (US)	M<br>F	1.05	1.04 – 1.06
Lung (UK)	M<br>F	0.60	0.56 – 0.65
Lung (US)	M<br>F	0.62	0.61 – 0.63
Pancreas (UK)	M<br>F	0.82	0.73 – 0.91
Pancreas (US)	M<br>F	0.88	0.88 – 0.89

Rectum (UK)	M<br>F	0.75	0.62 – 0.90
Rectum (US)	M<br>F	0.69	0.68 – 0.70
Small intestine (UK)	M<br>F	0.86	0.76 – 0.98
Small intestine (US)	M<br>F	0.90	0.89 – 0.91
Stomach (UK)	M<br>F	0.67	0.57 – 0.79
Stomach (US)	M<br>F	0.52	0.51 – 0.52

[1] Patient with missing data were excluded from statistical analyses.
